# Diagnostic delay in cluster headache: avenues for fixing the pathway

**DOI:** 10.1055/s-0046-1829151

**Published:** 2026-11-03

**Authors:** Welber Sousa Oliveira, Ivy Liger Riso, Mario F. P. Peres

**Affiliations:** 1 Centro Especializado em Hipermobilidade e Dor, Brasília DF, Brazil.; 2 Hospital Israelita Albert Einstein, Instituto do Cérebro, São Paulo SP, Brazil.; 3 Universidade de São Paulo, Faculdade de Medicina, Instituto de Psiquiatria, São Paulo SP, Brazil.

Few disorders in medicine combine such diagnostic clarity with such diagnostic failure. Cluster headache (CH) has among the most distinctive phenotypes in all of neurology — strictly unilateral, excruciating attacks of 15 to 180 minutes, clustered in time, with ipsilateral cranial autonomic features and near-pathognomonic restlessness. The criteria have been stable for decades. Yet in the study by Silva and colleagues published in this issue, Portuguese patients waited a mean of 6.4 years for diagnosis, nearly half more than four years, one patient thirty-nine. 1

Portugal does not perform poorly. Portugal performs typically. Comparable delays have been documented in the United States, the Netherlands, Belgium, and Spain, and Buture's systematic review placed the international range at 3.6 to 9.0 years 2 3 4 5 6 Twenty-five years separate the earliest of these studies from the present one, and the delay has not moved. This is the fact that should organize our response. Existing efforts have not produced consistent or durable improvement and simply repeating them is unlikely to be sufficient.

Part of the explanation is structural. CH affects roughly 124 per 100,000 individuals while its chronic form meets FDA and EMA thresholds for rare-disease designation. 7 Brazilian epidemiological data are consistent with this, and we have recently argued that CH qualifies as a rare disease in Brazil. 8 9 Rare diseases have a well-described natural history in health systems — diagnostic odysseys, low suspicion outside referral centers, no dedicated pathway. A rare disease with a common presentation is the worst case: prevalent enough to be mistaken for something else at every encounter, rare enough that no one is looking for it.

The Portuguese data show exactly this. Fifty-nine percent received an alternative diagnosis first, most often migraine, then sinusitis, trigeminal neuralgia, dental pathology. Prior triptan and topiramate use predicted longer delay. Most patients passed through two or more specialties. The first contact was general practice in a third of cases and emergency services in a fifth, with dentistry, ophthalmology, and otorhinolaryngology accounting for the remainder. The clinician recognized a severe primary headache and reached for the prevalent diagnosis without interrogating what distinguishes the rare one.

The authors call for physician education, cautious empirical prescribing, and optimized referral. All three are correct and all three are under-specified — and the first is precisely what has been attempted, without measurable effect, for a quarter century. Education without a pathway produces clinicians who suspect CH and have nowhere to send the patient. What follows is an attempt to specify the pathway.

A functioning referral and counter-referral system. The core deficit is architectural. There is no defined route from suspicion to specialist assessment, and no defined route back. Rare diseases require concentrated expertise. Headache centers should be formally identified as the destination for patients with suspected cluster headache. Existing referral regulation systems should provide fast-track pathways for these patients instead of routing them through routine general neurology waiting lists.

Once diagnosis and an initial treatment plan are established, ongoing management may return to the referring physician through a structured shared-care protocol, with clear guidance on verapamil titration, ECG monitoring, recognition of new bouts, and criteria for re-referral. The role of the specialist center is to establish the diagnosis, initiate evidence-based treatment, and support shared care, not to provide indefinite follow-up for every stable patient. Without an effective counter-referral pathway, specialist centers soon become overwhelmed with routine follow-up visits, making timely access for newly suspected patients increasingly difficult.

Recognition at the points of first contact. Recognition depends less on general awareness campaigns than on brief, protocol-embedded diagnostic prompts in the settings that the Portuguese data identify as the actual entry points—emergency departments, primary care, dentistry, ophthalmology, and otorhinolaryngology. Brief prompts addressing attack duration, restlessness, and ipsilateral cranial autonomic symptoms, embedded at the point of clinical decision-making, are likely to be more effective than stand-alone educational activities. Emergency departments deserve particular attention: one-fifth of first consultations occur there, and the encounter is brief, high-volume, and protocol-driven, making this setting particularly suitable for simple diagnostic prompts.

Repeated migraine-directed treatment in a patient with recurrent, strictly unilateral, short-lasting attacks should prompt diagnostic reconsideration rather than automatic escalation of migraine therapy. In the Portuguese cohort, prior use of triptans and topiramate was associated with longer diagnostic delay, probably reflecting prolonged treatment under an alternative diagnosis rather than an effect of the medications themselves.

Public campaigns may still have a role, particularly in encouraging earlier presentation and helping patients describe the timing, behavior, and autonomic features of their attacks. Once patients enter the healthcare system, however, timely diagnosis depends on how effectively clinicians recognize these features within routine care.

Access to essential therapies. Diagnosis without timely access to treatment is an incomplete pathway. Rapid referral achieves little if effective therapies are not readily available once the diagnosis is established. Reliable access to subcutaneous sumatriptan, verapamil, and bridging corticosteroids is an integral component of any cluster headache care pathway. Their inclusion in the 2025 WHO Model List of Essential Medicines underscores the importance of making evidence-based treatment available within functioning health systems. 10

Silva and colleagues have documented a failure mode that is global, persistent, and unresponsive to our current remedies. The next step is not broader awareness alone, but a care pathway that links early recognition, rapid specialist assessment, access to essential therapies, and structured return to longitudinal care. The remedy is not more information in the system. It is a pathway through it.
